# EMX2 gene expression predicts liver metastasis and survival in colorectal cancer

**DOI:** 10.1186/s12885-017-3556-2

**Published:** 2017-08-22

**Authors:** Berk Aykut, Markus Ochs, Praveen Radhakrishnan, Adrian Brill, Hermine Höcker, Sandra Schwarz, Daniel Weissinger, Roland Kehm, Yakup Kulu, Alexis Ulrich, Martin Schneider

**Affiliations:** 1 Department of General, Visceral and Transplantation Surgery, University of Heidelberg, Heidelberg University Hospital, Im Neuenheimer Feld 110, 69120 Heidelberg, Germany; 2 Department of Biotechnology, University of Heidelberg, Heidelberg, Germany

**Keywords:** Colorectal cancer, Homeobox gene, Adenoviral therapy, Univariate analyses, Metastasis

## Abstract

**Background:**

The Empty Spiracles Homeobox (EMX-) 2 gene has been associated with regulation of growth and differentiation in neuronal development. While recent studies provide evidence that EMX2 regulates tumorigenesis of various solid tumors, its role in colorectal cancer remains unknown. We aimed to assess the prognostic significance of EMX2 expression in stage III colorectal adenocarcinoma.

**Methods:**

Expression levels of EMX2 in human colorectal cancer and adjacent mucosa were assessed by qRT-PCR technology, and results were correlated with clinical and survival data. siRNA-mediated knockdown and adenoviral delivery-mediated overexpression of EMX2 were performed in order to investigate its effects on the migration of colorectal cancer cells in vitro.

**Results:**

Compared to corresponding healthy mucosa, colorectal tumor samples had decreased EMX2 expression levels. Furthermore, EMX2 down-regulation in colorectal cancer tissue was associated with distant metastasis (M1) and impaired overall patient survival. In vitro knockdown of EMX2 resulted in increased tumor cell migration. Conversely, overexpression of EMX2 led to an inhibition of tumor cell migration.

**Conclusions:**

EMX2 is frequently down-regulated in human colorectal cancer, and down-regulation of EMX2 is a prognostic marker for disease-free and overall survival. EMX2 might thus represent a promising therapeutic target in colorectal cancer.

## Background

Colorectal cancer is the third most common cancer in men and women and the third leading cause of cancer-related deaths in the western world [1]. While screening programs have led to a reduction in colorectal cancer mortality, there is considerable room for improvement in identifying prognostic markers that predict which patients are at risk for metastatic disease [2, 3]. International Union Against Cancer (UICC) stage III colorectal cancer is characterized by cancer spread to nearby lymph nodes and patients with stage III disease are generally at risk for recurrent disease or distant metastasis [4]. Therefore, most national guidelines recommend perioperative radiochemotherapy for management of patients with stage III colorectal cancer [5, 6].

The homeodomain-containing transcription factor EMX2 (Empty Spiracles Homeobox 2) belongs to the Homeobox gene family which encodes transcriptional regulatory proteins that are essential for growth and differentiation [7–9]. EMX2 plays a pivotal role during brain development and homozygous EMX2 mutations in mice are associated with ectopic Wnt expression resulting in cortical dysplasia [10–12]. Aberrant signaling of homeobox genes has been shown in many types of cancer [13]. Accordingly, recent studies suggest a possible involvement of EMX2 in several human cancers including lung, endometrial and gastric cancer [14–17]. Moreover, EMX2 has been shown to be a predictive marker for survival in lung cancer [18]. However, to our knowledge, no study has evaluated the role of EMX2 in colorectal cancer thus far. In this study, we analyzed the specific expression of EMX2 transcripts in colorectal cancer and corresponding healthy mucosa from 31 patients and investigated putative clinical correlations. Moreover, we applied over- and underexpression of EMX2 in vitro in order to assess its functional significance in colorectal cancer spread.

## Methods

### Patients

Tissue samples of primary colorectal adenocarcinoma and corresponding healthy mucosa from a series of 31 patients suffering International Union Against Cancer (UICC) stage III colorectal cancer were included in this study. Samples from 29 separate patients suffering stage IV colorectal cancer were used for additional expression analyses in liver metastases. All patients underwent surgery at the Department of General, Visceral and Transplantation Surgery, University of Heidelberg, Germany, between November 2005 and March 2013. Written informed consent was obtained from all patients. The study was approved by the local ethics committee. Clinical characteristics like gender, age at surgery, tumor location, histopathologic diagnosis including tumor, node, metastasis classification system and International Union Against Cancer (UICC) stage, R classification, perioperative radiochemotherapy and overall survival (time from operation up to death or last follow-up) were obtained from each patient. Exclusion criteria for tissue samples were a histopathological type that was not adenocarcinoma, patients who had synchronous metastasis or patients who had a histopathology-negative lymph node status.

### qRT-PCR

Total RNA was extracted using RNeasy Mini Kit (Qiagen, Hilden, Germany). cDNA synthesis and real-time PCR were performed with a first strand cDNA Synthesis Kit (Thermo Fisher, Karlsruhe, Germany) and LightCycler 480 SYBR Green I Master (Roche Diagnostic GmbH, Germany) using specific primers. Gene expression levels were normalized to the housekeeping gene GUS for each sample. Expression of transcript levels in human cancers were calculated as the level of gene expression in each sample relative to the level of gene expression in the adjacent normal mucosa using the comparative 2^-∆∆Ct^ method [19], whereby “overexpression” indicates overexpression relative to the adjacent normal mucosa, whereas “underexpression” indicates underexpression relative to the adjacent normal mucosa. Primer sequences were as follows: GUS (forward) 5’-GATCCACCTCTGATGTTCACTG-3′; GUS (reverse) 3′-TTTATTCCCCAGCACTCTCG-5′; EMX2 (forward) 5′-GCTTCTAAGGCTGGAACACG-3′; EMX2 (reverse) 3′-CCAGCTTCTGCCTTTTGAAC-5′.

### Cell culture, adenoviral infection and siRNA transfection

Human colorectal cancer cell lines DLD1 (ATCC® CCL-221™) and CaCo2 (ATCC® HTB-37™) were obtained from ATCC (Manassas, VA, USA) and maintained in basal medium supplemented with 10% FBS and 1% penicillin/streptomycin at 37 °C and 5% CO_2_. Cell lines were routinely tested for mycoplasma. All cell lines were free of contaminants. Adenovirus was used for restoring expression of EMX2. Adenovirus used to express EMX2 (Ad-EMX2) or control (Ad-Null) was purchased from Vector Biolabs (PA, USA). Conversely, for knockdown of EMX2, transfection of cells was performed with EMX2 siRNA using HiPerFect (both Qiagen, Hilden, Germany).

### Migration experiments

For migration assays, a modified Boyden chamber assay (Greiner Bio-one, Germany) was used. Migration inserts were coated with Matrigel (250 μg/mL; BD Biosciences) and FBS was used as a chemoattractant. Migrated cells were quantified after 24 h by dissolving cell-bound crystal violet in 10% acetic acid. Results were normalized to cell proliferation, which was determined in parallel using the cell proliferation reagent WST1 (Roche Diagnostic GmbH, Germany) according to the manufacturer’s instructions.

### Western blot

Whole cell lysates were prepared in RIPA lysis buffer (Merck Millipore, Germany). Anti-EMX2 antibody (ab174897, Abcam, 1:500) and anti-Vinculin antibody (ab18058, Abcam, 1:2000) was used to detect EMX2 and Vinculin, respectively. Horseradish peroxidase-conjugated goat anti-rabbit secondary antibody (sc-2004, Santa Cruz, 1:1000) and goat anti-mouse antibody (sc-2005, Santa Cruz, 1:2000), respectively, was used to label the primary antibodies. SuperSignal TM West Dura Extended Duration Substrate was used as the chemiluminescence substrate. Chemiluminescence images of the western blots were recorded using an ultra-sensitive camera detection platform from Fusion systems (Vilber Lourmat Deutschland GmbH). Semi-quantitative analyses of the resulting images were performed applying ImageJ software (National Institutes of Health, Bethesda, USA).

### Immunohistochemistry

For histological assessment of EMX2 expression, paraffin-embedded tissues were sectioned at 6 μm thickness, deparaffinized with xylene and rehydrated in a graded series of alcohols. For immunohistochemical staining, sections were blocked, and incubated overnight using a rabbit polyclonal anti-EMX2 primary antibody (Thermo Fisher, 1:200) at 4 °C in a humidified chamber. The slides were then treated with biotinylated horse anti-rabbit HRP conjugated secondary antibody (Vector Laboratories, 1:200). The sections were counterstained with hematoxylin. Images were captured using a Zeiss Axiostar Plus microscope equipped with an Axiocam MRC camera (Zeiss, Jena, Germany).

### Statistical analysis

Statistical analyses were conducted with Excel 2010 (Microsoft, Redmont, WA, USA) and SPSS version 21 (IBM, Armonk, NY, USA). Expressional changes were assessed by the two-tailed student’s t-test. Univariate analysis was performed using the log-rank test and Fisher’s exact test. The Kaplan-Meier method was used to estimate cancer-related survival. Differences between survival curves were evaluated by log-rank test. The Cox proportional hazard model was used to calculate survival related hazard ratios. Results were considered significant at a *P* value ≤0.05.

## Results

### Patient cohort

A total of 31 patients with International Union Against Cancer (UICC) stage III adenocarcinoma of the colon or rectum were included in the study. Patients with synchronous metastasis or who had a histopathology-negative lymph node status were excluded. Median age at the time of operation was 67 years. The median follow-up time was 1539 ± 155 days (range 452–3732 days). Eleven patients died of metastatic disease during follow-up. Mean overall survival of all patients was 54.4 months. Of the 31 cases, 15 patients presented with disease in the rectum at initial diagnosis; the remaining patients had a primary lesion in the colon. Each patient underwent surgical resection according to the localization of the tumor: 8 patients had low anterior resection, 7 high anterior resection, 2 abdominoperineal resection, 9 right colectomy, 2 left colectomy, 3 sigmoid colectomy. Table 1 lists patient and disease characteristics. Down-regulation of EMX2 was significantly associated with higher T and N-stage as well as metachronous liver metastases. Patients who suffered from liver metastasis (M1) had a significantly reduced overall survival.

**Table 1:** Correlation of clinical parameters of 31 patients with expression of EMX2 and overall survival

Characteristic	n	EXM2high(%)	EXM2low(%)	*P*-value (Fisher’s exact test)	Mean overall survival (months)	*P*-value (Log-rank test)
Gender						
Male	23	11(47,83%)	12(52,17%)	0,1552	55,2	0,7989
Female	8	3(37,50%)	5(62,50%)		52,1	
Age at operation						
< median = 67	17	7(41,18%)	10(58,82%)	0,2559	51,4	0,5648
< median = 67	14	7(50,00%)	7(50,00%)		57,7	
Localisation						
Colon	16	6(37,50%)	10(62,50%)	0,0649	54,9	0,9256
Rectum	15	8(53,33%)	7(46,67%)		53,9	
T stage						
T1	0	0	0	0,0413	N.A.	0,2123
T2	4	3(75,00%)	1(25,00%)		78,5	
T3	22	11(50,00%)	11(50,00%)		51,9	
T4	5	0(0,00%)	5(100%)		46,4	
N stage						
N1	17	9(52,94%)	8(47,06%)	0,0226	54,8	0,9444
N2	14	5(35,71%)	9(64,29%)		54,0	
Resection margin						
R0	31	14(45,16%)	17(54,84%)	N.A.	54,4	N.A
R1	0	0	0		N.A.	
R2	0	0	0		N.A.	
Rx	0	0	0		N.A.	
Occurrence of liver metastasis						
Yes	14	3(21,43%)	11(78,57%)	0.0001	43,0	0,0415
No	17	11(64,71%)	6(35,29%)		63,8	
Perioperative chemotherapy						
Yes	27	12(44,44%)	15(55,56%)	0,4788	52,6	0,3842
No	4	2(50,00%)	2(50,00%)		66,7	

*NA* not applicable

### Expression of EMX2 in colorectal cancer and corresponding healthy tissue

**Fig. 1:**
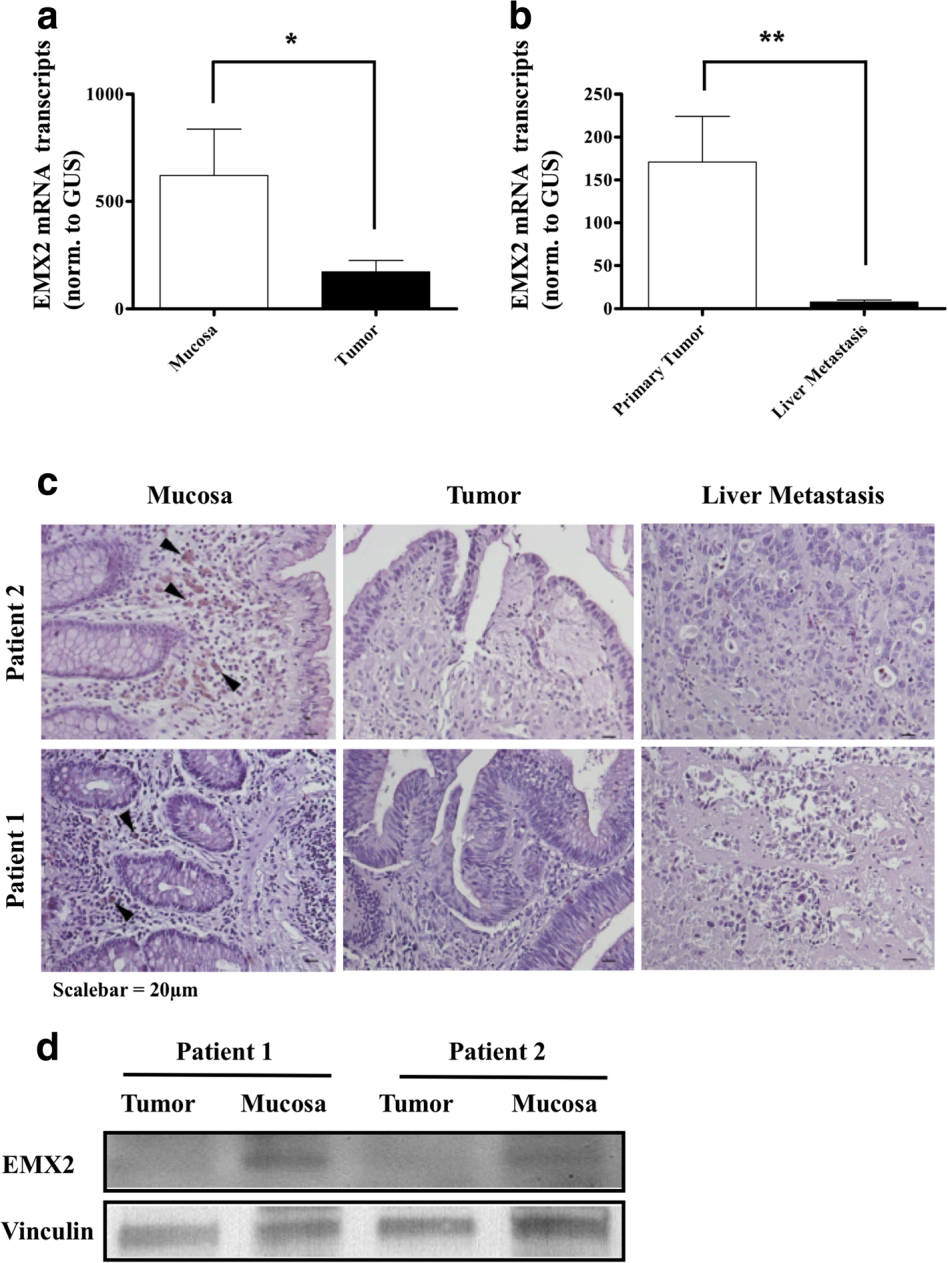
qRT-PCR depicting relative EMX2 mRNA expression in **a** primary stage III colorectal cancer samples and adjacent normal tissue. EMX2 levels in **b** liver metastases from CRC were further down-regulated compared to primary tumors. Bars indicate mean ± SEM (* *P* < 0.05, *n* = 31; ** *P* < 0.01, *n* = 31 primary tumors; 29 colorectal liver metastases). **c** Immunohistochemical staining, revealing EMX2 protein expression (arrowheads) in normal mucosa, but not in corresponding primary tumors or in liver metastases of each 2 representative patients. **d** Western Blot revealing EMX2 expression in primary tumor and normal mucosa from 2 representative patients

The expression of EMX2 transcripts was measured in paired normal mucosa and tumor tissue samples, revealing that EMX2 expression was significantly lower in colorectal tumor samples compared to their corresponding healthy mucosa (Fig. 1a). Next, we sought to investigate whether EMX2 was also down-regulated in colorectal liver metastases. For this purpose, EMX2 transcript expression levels were assessed in 29 colorectal liver metastases from our tissue biobank, and EMX2 expression in metastases was compared to expression levels in primary colorectal tumors. Indeed, EMX2 expression levels were further down-regulated in colorectal cancer liver metastases compared to primary tumor tissue from patients suffering stage III colorectal cancer (Fig. 1b). Given that EMX2 transcript levels were downregulated in primary colorectal cancer as well as colorectal liver metastases, we sought to determine protein levels of EMX2 by means of immunohistochemistry and Western blotting. In line with the transcript expression data outlined above, we could detect expression of EMX2 in healthy mucosa, but not in primary colorectal cancer samples or colorectal liver metastases (Fig. 1c,d). Taken together, these data suggest that down-regulation of EMX2 expression occurs in primary and, even more, in metastatic colorectal cancer.

### Down-regulation of EMX2 is associated with decreased survival in stage III patients

**Fig. 2:**
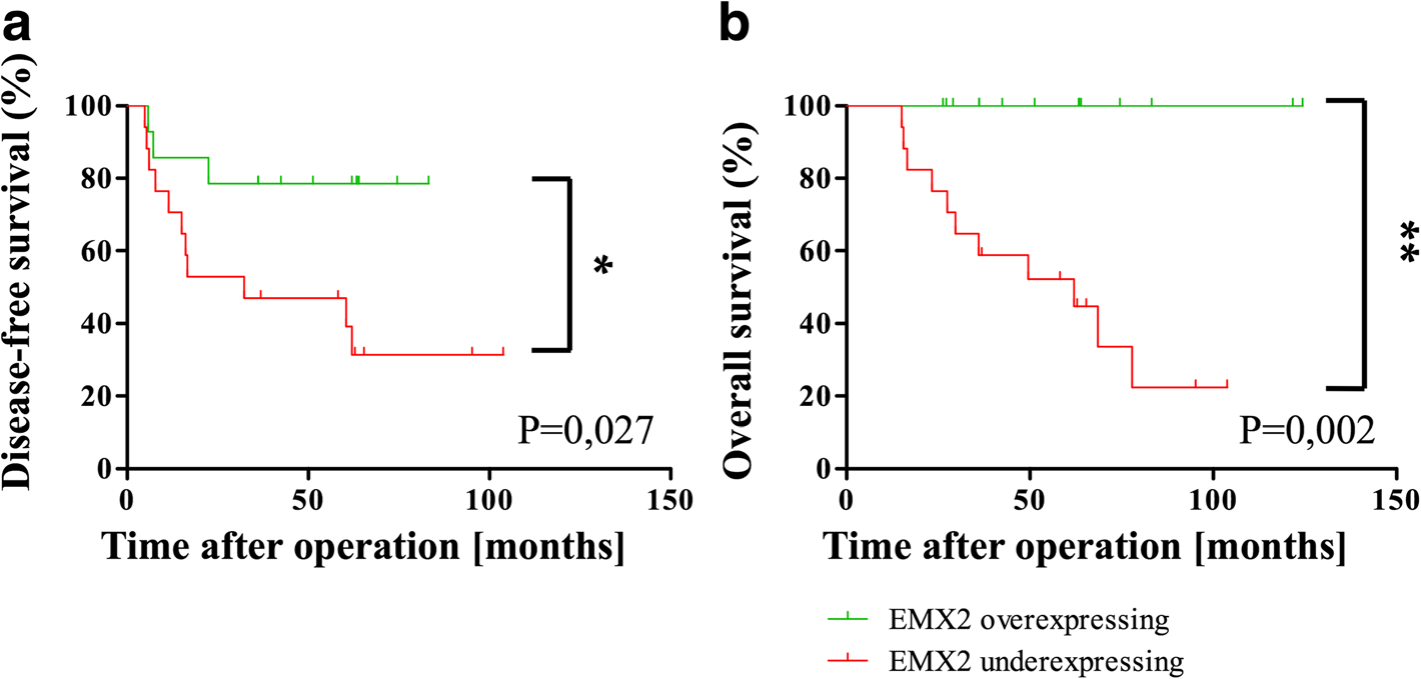
Correlation of EMX2 mRNA expression levels (tumor/mucosa) and **a** disease-free survival UICC III patents in univariate analysis (* *P* < 0.05, *n* = 31; hazard ratio 3.254; 95% confidence interval 1.137; 9.312). **b** Overall survival of UICC III assessed by Kaplan-Meier plot (** *P* < 0.001, *n* = 31; hazard ratio 6.619; 95% confidence interval 2.021; 21.68)

To investigate putative effects of down-regulated EMX2 expression on patient prognosis, we analyzed whether EMX2 transcript expression was associated with progression to metastatic disease. To measure expressional changes in our patient collective, we normalized EMX2 expression levels of primary tumors to those of their adjacent normal mucosa tissue. A comparison of patients harboring EMX2 over-expressing tumors to those carrying EMX2 under-expressing tumors was performed by Kaplan-Meier analysis using Cox proportional hazards modeling, and demonstrated a significant association between down-regulated tumoral EMX2 expression and the occurrence of colorectal liver metastases. Consistently, both disease free and overall survival were significantly decreased in patients displaying down-regulated EMX2 expression levels in their primary tumors (Fig. 2a,b). Taken together, these results indicate that decreased EMX2 expression predicts metastatic progression and unfavorable outcome in stage III colorectal cancer.

### Adenoviral delivery of EMX2 attenuates the migration of colorectal cancer cells

**Fig. 3:**
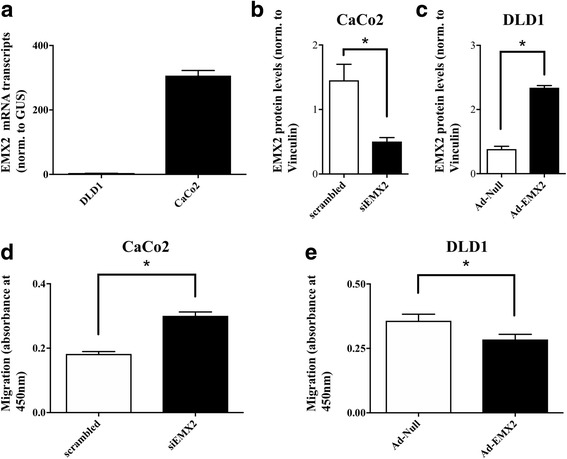
**a** qRT-PCR revealing EMX2 transcript expression in two different colorectal cancer cell lines (DLD1, CaCo2). **b** Semiquantitative analysis of immunoblotting revealing residual EMX2 expression after control-transfection versus siRNA-mediated EMX2 knockdown in CaCo2 cells and **c** Ad-Null versus Ad-EMX2-transfected DLD1 cells. Bars indicate mean ± SEM. **d** Transwell migration assay revealing enhanced migratory potential of CaCo2 cells upon siRNA-mediated knockdown of EMX2 compared to control-transfected (scrambled) cells. Restoration of EMX2 expression in **e** DLD1 cells using an adenoviral delivery system resulted in attenuated migration. Ad-Null and Ad-EMX2 represent the empty adenoviral vector control and the adenoviral vector containing EMX2 cDNA, respectively. *Bars* indicate mean ± SEM of three independent experiments (* *P* < 0.05)

In an attempt to further elucidate whether enhanced metastatic progression of colorectal tumors could indeed be attributable to expressional changes of EMX2, we examined the effects of EMX2 on the migratory potential of colorectal tumor cells in vitro. In search for a suitable in vitro model, imitating up- or down-regulation of EMX2, we screened various colorectal cancer cell lines for their EMX2 expression levels. While DLD1 cells were found to lack EMX2, CaCo2 cells displayed sustained expression of EMX2 (Fig. 3a). We therefore performed siRNA-mediated EMX2 knockdown in CaCo2 cells, which significantly reduced EMX2 expression on the transcript level (EMX2 mRNA, norm. to GUS: 678.0 ± 43.41 in control-transfected cells versus 438.0 ± 26.53 in siEMX2-transfected cells; *P* < 0.05; *n* = 3), as well as on the protein level (Fig. 3b). Intriguingly, tumor cell migration was significantly increased upon knockdown of EMX2 in this cell line (Fig. 3d). We likewise examined the significance of EMX2 in cell migration by infection of DLD1 cells (which display low EMX2 expression levels at normal baseline conditions) with an adenovirus expressing EMX2 (Ad-EMX2), or with an empty vector control (Ad-Null). Transfection with Ad-EMX2 caused robust and stable over-expression of EMX2 in DLD1 cells on the transcript level (EMX2 mRNA, norm. to GUS: 3.9 ± 0.3 in Ad-Null-transfected cells versus 3466.654 ± 840.82 in Ad-EMX-2-transfected cells; *P* < 0.001; *n* = 3), as well as on the protein level (Fig. 3c). Consistent with our results obtained from CaCo2 cells, adenoviral overexpression of EMX2 resulted in significantly decreased migration of DLD1 tumor cells (Fig. 3e). Collectively, these in vitro data confirm the notion that EMX2 down-regulation in colorectal cancer cells affects their migratory potential, thus contributing to an increased rate of distant metastasis and unfavorable outcome.

## Discussion

The homeodomain-containing transcription factor EMX2 was first described as an important mediator in embryonic development as EMX2 has been shown to play a role in neuroblast proliferation, migration and differentiation [20–22]. While transcriptional targets of EMX2 remain largely unidentified, loss of EMX2 function is associated with impaired development of the cortex [23–25]. Moreover, homozygously EMX2-deficient mice have been shown to display ectopic Wnt expression [12]. Wnt is an oncogene and its signaling represents an early event in a majority of colorectal cancers [26]. Wnt pathway signaling has also been associated with metastatic spread and stemness in CRC [27–29].

Recently, the role of EMX2 has been explored in various solid tumors. Several lines of evidence suggest that EMX2 is down-regulated in lung cancer [14, 30, 31]. While the mode of EMX2 down-regulation in non small-cell lung cancer (NSCLC) has been identified as epigenetic silencing, restoration of EMX2 has been shown to antagonize Wnt and to restore sensitivity to cisplatin [14]. More recently, loss of EMX2 expression has been demonstrated in gastric cancer cell lines and primary gastric cancer. EMX2 down-regulation was associated with promoter hypermethylation and the adenoviral delivery of EMX2 in a mouse model of gastric cancer significantly suppressed tumor growth [16]. Thus, these previous observations support our present finding that down-regulation of EMX2 has protumorigenic effects in colorectal cancer.

To our knowledge, this is the first investigation of the functional role of EMX2 in colorectal cancer. In our patient collective, EMX2 was frequently down-regulated in tumor tissue in comparison to matched normal mucosa samples. Down-regulation of EMX2 was associated with metastatic tumor progression and decreased overall survival. These results differ from a previous study, where loss of EMX2 was found in only 2–5% of colorectal cancers [32]. In contrast to the latter study, we found a down-regulation of EMX2 in 54% of our patient collective. These observed differences can in part be explained by the different methods that were applied to assess EMX2 expression. While Kim et al. used immunohistochemical analysis, we applied qRT-PCR to detect EMX2 expression levels [32]. We then normalized expression levels of EMX2 in tumors to expression levels in adjacent normal mucosa. We believe that qRT-PCR is a robust and sensitive tool for quantification of gene expression levels.

Nevertheless, our study has several limitations. First, the power to make statistical inferences was limited by a modest sample size. The main reason for this is the limited number of cases where both tumor and normal mucosa samples were available for RNA extraction, along with appropriate follow-up data allowing for the detection of metastatic disease and assessment of survival. Second, although colorectal liver metastases showed a further decrease in EMX2 expression levels when compared to primary colorectal cancer samples, it remains unknown whether this is due to an evolutionary decrease of EMX2 expression from primary tumor to metastasis and therefore a possible driver or prerequisite for metastatic outgrowth or whether this is a mere coincidence. Metastatic progression of cancer is a complex process involving a multi-step process, where migration represents a key element in the process of the metastasic cascade [33]. The functional assays in this study assessing tumor cell migration suggest a potential role for EMX2 in metastatic disease progression since EMX2 knockdown resulted in increased migration while restoration of EMX2 using an adenoviral vector led to decreased migration. We used a recombinant human adenovirus type 5 as delivery system since this is the vector of choice for functional genomics research [34]. While there are still many challenges that need to be overcome before adenoviral vectors can be safely used in cancer patients, adenoviral vector-based therapeutic strategies represent a promising tool for cancer gene therapy [35, 36]. Altogether the role of EMX2 expression in metastatic spread would be a valuable area of future research in a larger patient cohort.

In summary, our data encourage a significant role of EMX2 in the progression and metastasis of colorectal cancer. Our study demonstrates that a low EMX2 expression level is an independent prognostic factor and correlates with dismal prognosis, decreased overall survival and the development of metastatic disease in stage III colorectal cancer patients. Thus, the study at hand provides a first evidence for the role of EMX2 as a suppressor of metastasis in colorectal cancer. Further, we provide evidence that EMX2 has predictive value as a prognostic factor in stage III colorectal cancer as well as a possible functional role in metastatic spread. Therefore, restoration of EMX2 via gene therapy may represent a promising therapeutic strategy for tailored anticancer therapy.

## Conclusions

EMX2 is frequently down-regulated in both primary colorectal cancer and colorectal cancer liver metastases. Down-regulated EMX2 is a strong predictor for shortened disease-free and overall survival in stage III colorectal cancer. In vitro knockdown of EMX2 leads to increased tumor cell migration while adenoviral restoration of EMX2 is associated with decreased migration. EMX2 might represent a promising molecular target for colorectal cancer therapy.

## Abbreviations

CRC: colorectal cancer
EMX2: Empty Spiracles Homeobox 2
GUS: β-Glucuronidase
NSCLC: Non-small cellular lung cancer
qRT-PCR: Real-Time Quantitative Reverse Transcription PCR
SEM: Standard error of the mean
UICC: International Union Against Cancer
